# 

**Published:** 1888-11

**Authors:** 


CONTENTS:

ARTICLE I.—Georgia State Dental Society. By Mrs. M. W. J ... .... 289

" II.—The Relation of Social Life to Surgical Disease. By D. Hayes Agnew, M. D ........ 305

" III.—The Implantation of Teeth in Artificial Sockets. By Dr. G. W. Weld ........ 319

" IV.—A Few Items of Importance. By Dr. E. Parmley Brown 323

EDITORIAL.

University of Maryland Dental Department ........ 327

Curious Fatality Which Pursues the First-Born Sons of the Crockett Family ........ 327

Toothmarks Made by a Burglar Lead to His Capture ........ 329

Death While Under the Influence of Nitrous Oxide Gas........ 330

MONTHLY SUMMARY.

Effects of Mental Overwork ........ 331

Do all Amalgams Shrink? ........ 332

The Glands ........ 333

Cigarette Smoking ........ 334

Where do Teeth Decay? ........ 335

A Wonderful Germicide ........ 335

Causes of Mortality ........ 336

Detection of Concealed Insanity by the Use of Nitrous Oxide ........ 336

